# Impacts of genetic correlation on the independent evolution of body mass and skeletal size in mammals

**DOI:** 10.1186/s12862-014-0258-0

**Published:** 2014-12-14

**Authors:** Marta Marchini, Leah M Sparrow, Miranda N Cosman, Alexandra Dowhanik, Carsten B Krueger, Benedikt Hallgrimsson, Campbell Rolian

**Affiliations:** Department of Comparative Biology and Experimental Medicine, Faculty of Veterinary Medicine, University of Calgary, 3330 Hospital Drive NW, Calgary, AB T2N4N1 Canada; Department of Anatomy and Cell Biology, Faculty of Medicine, University of Calgary, 3330 Hospital Drive NW, Calgary, AB T2N4N1 Canada

**Keywords:** Evolvability, Genetic lines of least resistance, Limb length, Body mass, Allometry, Artificial selection, Rodent locomotion

## Abstract

**Background:**

Mammals show a predictable scaling relationship between limb bone size and body mass. This relationship has a genetic basis which likely evolved via natural selection, but it is unclear how much the genetic correlation between these traits in turn impacts their capacity to evolve independently. We selectively bred laboratory mice for increases in tibia length independent of body mass, to test the hypothesis that a genetic correlation with body mass constrains evolutionary change in tibia length.

**Results:**

Over 14 generations, we produced mean tibia length increases of 9-13%, while mean body mass was unchanged, in selectively bred mice and random-bred controls. Using evolutionary scenarios with different selection and quantitative genetic parameters, we also found that this genetic correlation impedes the rate of evolutionary change in both traits, slowing increases in tibia length while preventing decreases in body mass, despite the latter’s negative effect on fitness.

**Conclusions:**

Overall, results from this ongoing selection experiment suggest that parallel evolution of relatively longer hind limbs among rodents, for example in the context of strong competition for resources and niche partitioning in heterogeneous environments, may have occurred very rapidly on geological timescales, in spite of a moderately strong genetic correlation between tibia length and body mass.

**Electronic supplementary material:**

The online version of this article (doi:10.1186/s12862-014-0258-0) contains supplementary material, which is available to authorized users.

## Background

**Figure 1:**
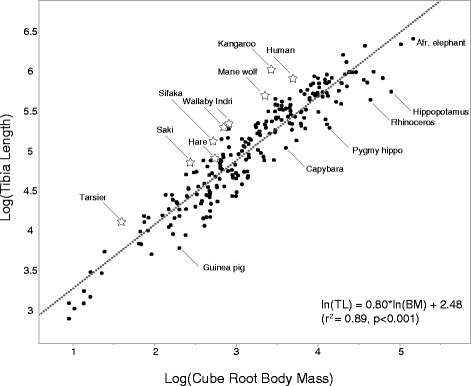
**Scaling relationship between cube root body mass and tibia length in mammals.** Ordinary least squares regression of log tibia length (mm) on log cube root body mass (g^0.33^) across 227 species of terrestrial mammal, from the ~20 g red-backed vole (*Myodes gapperi*) to the 5.4 ton African elephant (*Loxodonta africana*). There is a negative allometric relationship between body mass and tibia length; species that deviate the most from the expected relationship tend to be specialized for bounding, cursoriality and/or bipedal locomotion (positive residuals, a sample of better known species with large positive residuals is indicated by stars), or for graviportal locomotion (negative residuals). Data from [5,13-16], Campbell Rolian (personal data) and Kevin Middleton (personal data).

Limb bone size and shape among terrestrial mammals scale allometrically with body mass, both within populations [1], and also across species with a wide range of body masses [2-6] (Figure 1). For example, limb bone lengths and diameters tend to be proportional to the cube root of body mass among terrestrial quadrupeds [3]. The scaling of limb bone dimensions with body mass ensures proper musculoskeletal function at different body sizes. Appropriate scaling of limb bone, length, diameter and cross-sectional dimensions with increasing body mass prevents bones from failing under increased gravitational and locomotor loads [7-9]. Moreover, the near-geometric scaling of limb bone length among geometrically similar terrestrial mammals may also maintain the mass-specific metabolic cost of locomotion within similar ranges at different body size [10]. These scaling relationships suggest that significant mismatches between body mass and limb bone dimensions, within populations, can have negative impacts on evolutionary fitness, and should be selected against. In other words, the positive phenotypic correlation of skeletal dimensions to body mass in terrestrial mammals - which has a genetic basis [11,12] - likely evolved by means of natural selection.

The links between limb bone dimensions and body mass indicates that they are functionally, developmentally and genetically integrated [17], to the extent that selection on body mass causes correlated changes in the size and shape of individual bones, and vice versa. However, limb size in some terrestrial mammals also deviates from the predicted relationship between limb bone morphology and body mass (Figure 1). Importantly, these macroevolutionary changes have occurred primarily in the context of functional specializations related to locomotion. One striking example of this can be seen in the relative length of the hind limb bones in species which have evolved specialized modes of locomotion such as hopping (e.g., kangaroos, jerboas), leaping (e.g., tarsiers, lemurs), bounding (e.g., macropods) and bipedal walking/running (humans). These species rely primarily on their hind limbs for propulsion during locomotion, and it is thought that their relatively longer hind limb bones improve whole organism performance during locomotion by reducing the metabolic cost of transport [18-20].

The fact that increased relative hind limb bone length has evolved repeatedly, and convergently, in these functionally specialized terrestrial mammals clearly shows that skeletal length can evolve adaptively independently of body mass, despite the existence of a strong phenotypic and genetic correlation between these complex traits. What is less clear, however, is how much phenotypic and genetic correlations with body mass impose constraints on the evolution of novel limb bone size and shape in mammals. For example, how much does the genetic correlation between body mass and hind limb bone length affect the magnitude and/or rate of evolutionary change in the latter?

### Genetic correlations and evolutionary constraints

Genetic constraints on the adaptive evolution of complex traits have been the subject of many theoretical and empirical studies (reviewed in [21,22]). These studies are based on the premise that the structure of phenotypic and genetic variance/covariance among multiple traits in a population can slow evolution towards optimally adapted phenotypes, depending on where this new optimum is located in multivariate space, and on the magnitude and direction of selection. For example, for two traits, adaptive evolution is thought to be most rapid along the direction of maximum genetic variance, which typically coincides with the direction of the phenotypic correlation between the traits. This direction, known as **g**_**max**_, represents a “line of least evolutionary resistance” (LLR) along which phenotypic change is “easiest” and the multivariate phenotype is most evolvable [23]. In contrast, the direction of least genetic variance, which is perpendicular to **g**_**max**_, is the line of greatest resistance to evolutionary change, i.e., where constraints imposed by genetic correlations among traits have their greatest impacts on adaptive evolution.

The potential constraining effects of genetic correlations among traits on their ability to respond to selection (their evolvability) is embodied in the **G**-matrix, the matrix of additive genetic variances and covariances between the traits [24]. The **G**-matrix not only describes how much additive genetic variance is present in a given trait, but also how much of this variance is tied up as covariance with other trait(s). The magnitude of additive genetic variance of the traits determines if and how they can evolve in response to directional selection. More importantly, the magnitude of the genetic covariance determines whether these traits can evolve independently in response to selection. When all the additive genetic variance of a trait is “locked up” as genetic covariance with another trait (i.e., the traits have a genetic correlation of 1), the traits are in theory not capable of independent evolution in response to directional selection [21,25,26]. A strong genetic correlation also means that, even if one of the traits is not under selection, it may co-evolve with another under selection [27].

Quantitatively, the impact of additive genetic variances and covariances on the response of multiple traits to selection is described by the following equation:

1 $$ \varDelta \overline{z}=G\beta $$

Where $\varDelta \overline{z}$ is a vector describing the change in the population means of the traits after an episode of selection, **G** is the additive genetic variance/covariance matrix, and *β* is a vector of selection gradients, the partial regression coefficients of the phenotypes on relative fitness [25,28]. Equation 1 summarizes and predicts the generational responses to selection in multiple traits as a function of the amount of genetic variance in each trait and the strength of the genetic covariance between them.

Empirically, the impact of genetic correlations embodied in the **G**-matrix on the evolvability of phenotypes has been studied using two complementary approaches. In the first approach, the constraining effect of the correlation between traits is assessed using artificial selection, by imposing selection regimes that would “push” the phenotype in multivariate space at right or near-right angles to **g**_**max**_, i.e., along the hypothetical line of greatest evolutionary resistance (reviewed in [21]). Owing in large part to the labor-intensive nature of selection experiments, such studies have been done almost exclusively on organisms that are short-lived, breed easily, and produce relatively large numbers of offspring, such as plants (e.g., *Arabidopsis,* [29], wild radishes, [21,30]) and insects (e.g., butterflies, [31-34], beetles [35], *Drosophila,* [36]). Although most studies show that evolution at right angles from **g**_**max**_ is possible, despite strong genetic correlations, a few have shown the existence of seemingly unbreakable, developmentally based constraints on the short-term evolution of traits perpendicular to **g**_**max**_ (e.g., color variation between eyespots in the wings of the butterfly *Bicyclus anynana* [34,37,38].

In the second approach, macroevolutionary patterns of covariance among traits are recorded across taxa, as illustrated in Figure 1 between body mass and tibia length. Following this, an estimate of the **G** matrix, typically based on its phenotypic counterpart (**P**), is used to determine how often taxa within specific radiations have diverged from the inferred LLR (e.g., [23,39-41]). These macroevolutionary studies have the advantage that they can be done with any group of organisms in which morphology and its underlying phenotypic or genetic covariance structure can be measured (e.g., vertebrates), and can even include fossils as a window into past morphological diversification, something which is not usually possible with plants and most invertebrates. More often than not, these studies show that diversification across related taxa occurs along LLRs, and departures from them are rarer [39,41]. For example, Renaud et al. (2006) [41] use major variation axes of the **P** matrix as substitutes for **G**, and major evolutionary transitions documented in the rodent fossil record as substitutes for $\varDelta \overline{z}$ to show that *Stephanomys,* a genus which evolved a highly specialized tooth morphology, departs from the expected alignment with the LLR describing (co)variance in tooth shape across taxa. The authors suggest that this unique tooth morphology evolved via climate-related selection as an adaptation for eating grass, in the context of the evolution of grasslands in southwestern Europe in the Miocene.

Due in part to the practical reasons outlined above, artificial selection experiments specifically investigating the evolvability of genetically correlated pairs of traits have not previously been done in vertebrates. Such artificial selection experiment could offer insights into the mechanisms of adaptive morphological evolution in mammals at macroevolutionary scales, for example with respect to the magnitude and direction of natural selection necessary to overcome any internal genetic constraints and produce adaptive changes in limb bone length associated with specialized modes of locomotion such as hopping or bipedalism. In this study, we report on the first 14 generations of a selective breeding experiment in which we bred laboratory mice for increases in tibia length independent of body mass. The establishment of this unique line of mice, hereafter the Longshanks mouse, is the foundation of a long-term experiment examining the microevolution of complex skeletal traits in vertebrates. Here, we test the general hypothesis that the genetic correlation between body mass and limb bone dimensions constrains the rate of evolutionary change in the latter. First, we report on the actual gains in relative tibia length achieved after 14 generations of selective breeding. Second, we determine to what degree the genetic covariance between tibia length and body mass has potentially and actually constrained the evolution of both traits within the experiment.

## Results

### Changes in tibia length and body mass due to selection

**Figure 2:**
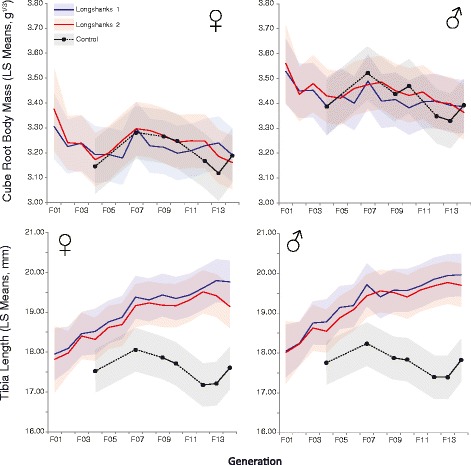
**Evolutionary changes in body mass and tibia length.** Least squared means of cube root body mass (top) and tibia length (bottom) by sex over 14 generations of selective breeding for increased tibia length in Longshanks 1 (blue), Longshanks 2 (red) and control mice (black). LS means are adjusted for covariation with litter size and age at measurement. Shaded areas indicate standard deviations. Control mice were measured only in generations indicated by a solid black circle. Dotted lines in the control mice represent missing values from generations in which the control line was not measured.

Figure 2 shows the changes due to selection for increases in relative tibia length in two independent lines of Longshanks mice (Lines 1 and 2, see Methods) compared with a control cohort (Line C). On average, tibia length increased by 0.67-0.94% per generation of selective breeding in males and females for both lines (Figure 2, Table 1). Tibia lengths in males and females are approximately equal, but females are substantially lighter than males, and accordingly have relatively longer tibiae (Table 1, Additional file 1: Table S1 and Additional file 2: Table S2). Figure 2 shows that the LS means for both body mass in generation F01 were substantially greater than in F02, because F01 mice were three weeks older and were fed a high-fat diet (see Methods). Further, in several generations, both tibia length and body mass decreased in the Longshanks lines. This decrease was not due to differences in the magnitude and direction of selection differentials or selection gradients (see below), but is likely an environmental artifact, perhaps due to stochastic or seasonal fluctuations in the housing conditions of the mouse colonies.

**Table 1:** **Least square means and standard deviations (SD) for cube root body mass (BM) and tibia length (TL) at F14 in males and females from all three lines, and percentage difference in Longshanks vs Control lines for both traits**

**Sex**	**Line**	**N**	**BM (SD)**	***%Δ (Long. – Ctrl.)***	**TL (SD)**	***%Δ (Long. – Ctrl.)***
**F**	1	73	3.188 (0.106)	+0.85	19.74 (0.97)	+12.79
	2	50	3.168 (0.107)	+0.20	19.14 (0.98)	+9.37
	C	42	3.188 (0.112)	-	17.5 (1.02)	-
**M**	1	65	3.387 (0.106)	+0.54	19.94 (0.96)	+12.53
	2	80	3.364 (0.111)	+0.28	19.71 (1.01)	+11.25
	C	42	3.392 (0.112)	-	17.72 (1.02)	-

**Figure 3:**
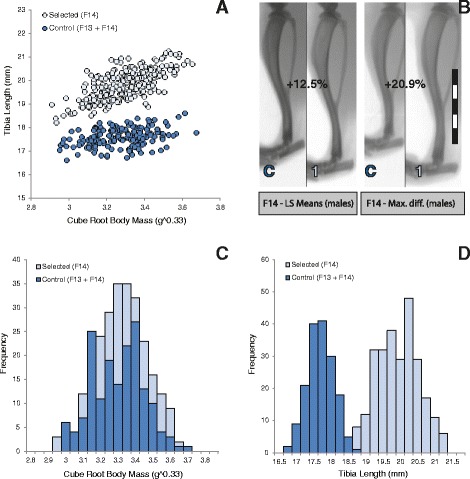
**Phenotypic differences in body mass and tibia length after 14 generations. A** (upper left): Scatterplot of tibia length (mm) on cube root body mass (g^0.33) for Lines 1 and 2 (data pooled, light blue circles) and Line C (dark blue circles). Data for Lines 1 and 2 from F14 (n = 264), Line C data include specimens from F13 and F14 (n = 167). **B** (upper right): the left image shows Line 1 and Line C individuals representing the observed mean tibia lengths for males in both lines, the right image shows an extreme difference between two males of identical age and body mass. Numbers represent the percentage increase in tibia length in Line 1 vs Line C. Scale bar = 1 mm. **C** (lower left): frequency distribution of cube root body mass for selected (light blue) and control lines (dark blue). **D** (lower right): frequency distribution of tibia length for selected (light blue) and control lines (dark blue). Only nine control individuals had longer tibiae than the selectively bred mouse with the shortest tibia.

The ANCOVA was performed with data from F14 only, and the LS means are adjusted for covariation with litter size and age at measurement. (see also Figures 2 and 3, Additional file 1: Table S1).

In both sexes, the cumulative increase in tibia length in the Longshanks mice relative to controls over the first 14 generations is equivalent to ~3.5 units of phenotypic standard deviation (the average standard deviation for tibia length by sex and line across generations F02-F14 was 0.54, see Additional file 1: Table S1), with observed mean increases of 9.4 to 12.8% (Table 1), but up to 20% between Longshanks and control individuals of the same age and mass (Figure 3). In contrast, cube root body mass fluctuates over the course of the 14 generations, but it is not significantly different between the lines at F14, for either sex (ANCOVAs controlling for litter size and age, F_2,164_ = 1.49, p = 0.23 for females, F_2,186_ = 0.22, p = 0.80 for males). At F14, frequency distributions of both traits between Longshanks and control mice (pooled by sex) show little overlap between tibia length, but near-perfect overlap in body masses between the groups (Figure 3).

### Selection parameters and G-matrices

**Figure 4:**
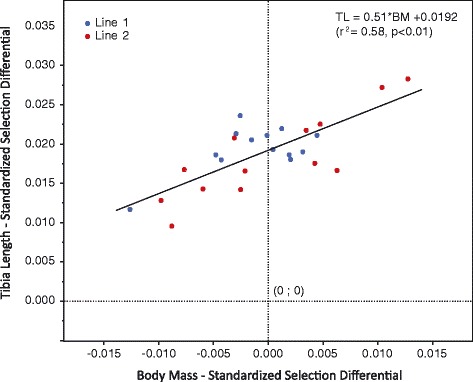
**Correlation between selection differentials.** Linear regression of the mean-standardized selection differential of tibia length on the mean-standardized selection differential for cube root body mass (see Additional file 1: Table S1). Data from Lines 1 (blue) and 2 (red) are pooled.

**Figure 5:**
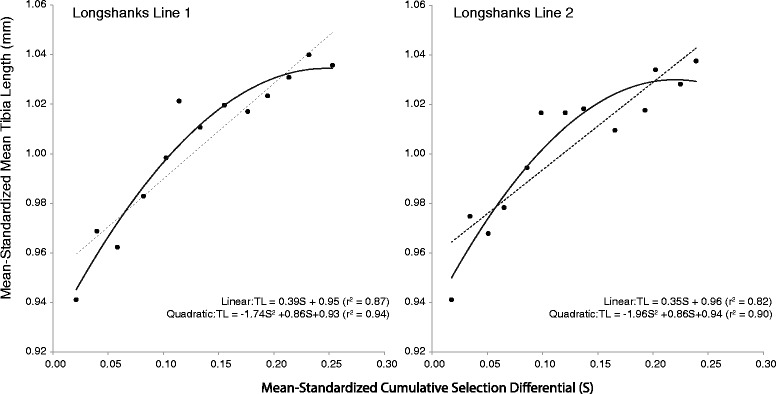
**Cumulative selection response in tibia length.** The cumulative response in tibia length (expressed as sample means each generation) is plotted as a function of the cumulative selection differential in Longshanks Line 1 (left) and Line 2 (right). Dashed lines correspond to linear regression models fitted to the data, solid curves correspond to polynomial regression models with both linear and quadratic terms. All coefficients in both models are significant at the p < 0.001 level.

Directional selection differentials were always much greater for tibia length than for body mass (Figure 4, Additional file 3: Table S3). For tibia length, the average selection differential across generations and lines is ~2% of the mean tibia length in a given generation. In contrast, for body mass the average is near zero, and in multiple generations it is negative. Despite this difference in magnitude, there is a significant positive correlation between the selection differentials for body mass and tibia length (Figure 4, r^2^ = 0.58, p <0.01). In other words, when the difference in tibia length between the breeders and the whole population in a given generation is greater, so too is the difference in body mass between the two groups. When the observed Longshanks means for tibia length in each generation are plotted against the cumulative selection differentials, the result is a significantly linear response for tibia length in the direction of selection (Figure 5). Note that the quadratic term for the polynomial regression model is also significant and negative in both Longshanks lines. Future generations of selection may help to determine whether this is a trend that reflects a slowing of the selection response by generation F13-F14, or whether it is a stochastic phenomenon. Finally, selection gradients acting on body mass are all negative, and positive in tibia length (Additional file 3: Table S3), with the average inferred strength of directional selection on tibia length being over two times greater, in absolute terms, than for body mass. The two traits are thus under opposite selection pressures, with tibia length experiencing relatively greater directional selection pressures.

**G** matrices were estimated with high confidence in both selected lines, and both are similar in magnitudes and error ranges (Additional file 4: Table S4). The results of the animal model analyses indicate that additive genetic variance accounts for 44% and 39% of phenotypic variance in body mass in Longshanks Lines 1 and 2, respectively (Additional file 5: Table S5, Additional file 6: Table S6 and Additional file 7: Table S7). For tibia length, additive genetic variance accounts for 58% of phenotypic variance in Line 1, while in Line 2 it is 58%. This translates into narrow-sense heritabilities of ~0.4 for body mass, and ~0.5 for tibia length in both lines. Genetic correlations are 0.40 and 0.48 in Lines 1 and 2, respectively, while their respective phenotypic correlations are 0.54 and 0.53 (Additional file 5: Table S5, Additional file 6: Table S6 and Additional file 7: Table S7) [11].

The dot products of the vectors of selection gradients and the first eigenvector of the **G** matrix (pointing in the positive direction) provide an estimate of the angle between the line of greatest genetic variance (**g**_**max**_) and the direction of selection. For Line 1, the mean angle is 67.3° ± 5.0° (SD), and ranges from 55 to 74°. In Line 2, the mean angle is 79.9° ± 5.9°, ranging from 70.0 to 90.8°. Thus, the dual ranking system we used to identify breeders in each generation produced selection gradients that departed substantially from the orientation of **g**_**max**_, and were often at near-right angles to it, i.e., along the direction expected to produce the greatest evolutionary constraints on independent change in both traits.

### Divergence of observed and predicted responses

**Figure 6:**
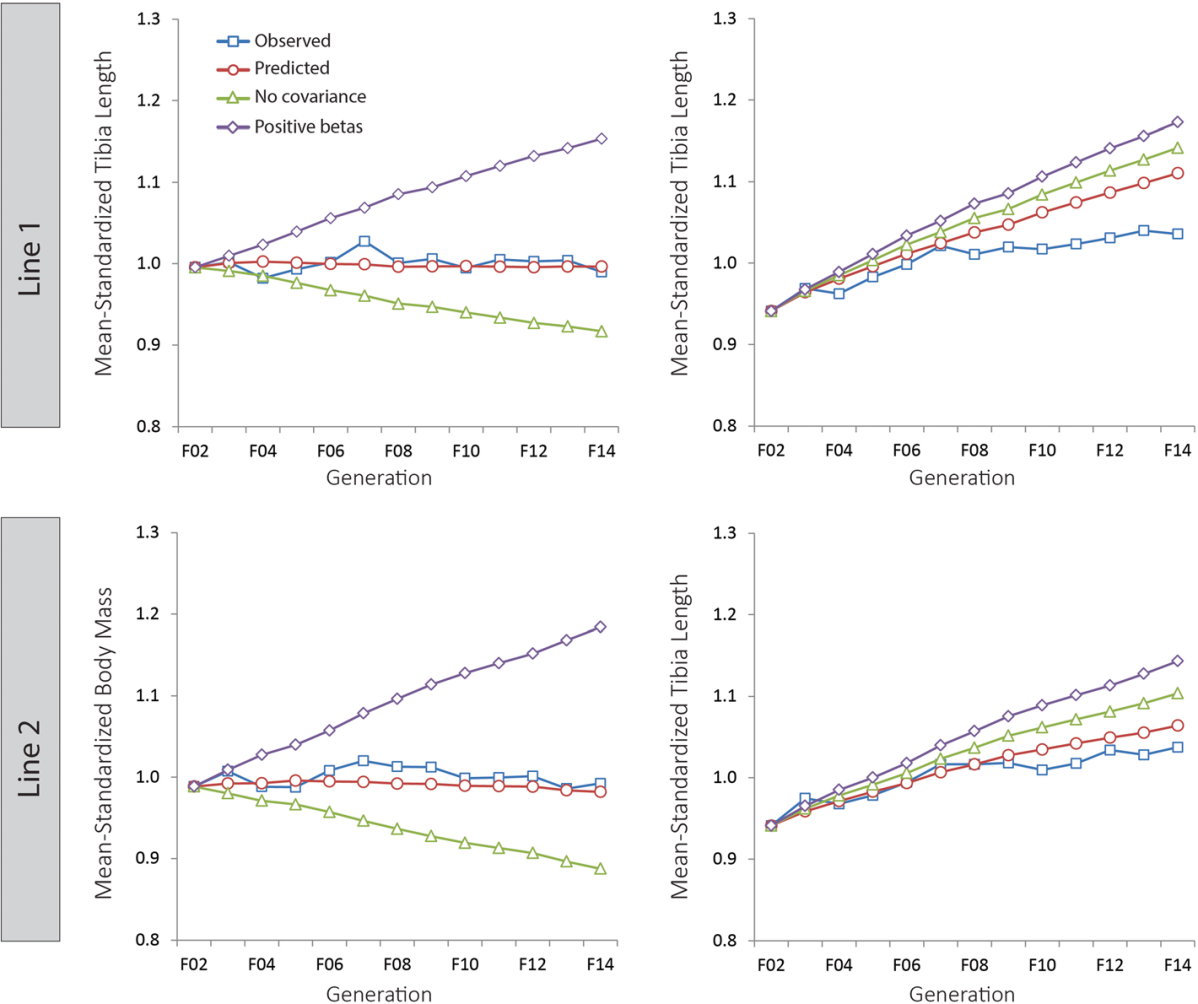
**Observed and predicted changes in body mass and tibia length under different evolutionary scenarios.** Observed (open red circles) and predicted (open blue squares) changes in cube root body mass (left panels) and tibia length (right panels) for Line 1 (top) and Line 2 (bottom) from generations F02 to F14. The green line (open triangles) shows the predicted changes in both traits in the absence of genetic covariance, while the purple line (open diamonds) shows the predicated changes in both traits if selection gradients were in the same (positive) direction.

Figure 6 shows the cumulative divergence between the predicted and observed changes in body mass and tibia length over generations F02-F14 (Table 2). The predicted changes in body mass are flat, and although there are fluctuations between generations, by generation F14, the observed body masses are only +0.1 and −1.4% off the predicted body masses in Lines 1 and 2, respectively. In contrast, the cumulative predicted gains for tibia length over 14 generations are 30-50% greater than the cumulative observed responses (15.0% vs 10.1% for Line 1, 13.1% vs 10.2% for Line 2, see Table 2). Much of this “missing” tibia length at F14 may be due to earlier generations in which responses in both body mass and tibia length were negative (Figure 2). By generation F14, this missing tibia length causes total tibia length to be 7.2 and 2.7% shorter than predicted in Lines 1 and 2, respectively (Table 2).

**Table 2:** **Observed and predicted changes in the mean-standardized mean cube root body mass (top) and tibia length (bottom) in Lines 1 and 2 after 14 generations of selection**

	**Line 1 (pooled sexes)**			**Line 2 (pooled sexes)**		
**Cube root body mass**	**Standardized mean**	**% diff. over F02**	**% diff. over F14 observed mean**	**Standardized mean**	**% diff. over F02**	**% diff. over F14 observed mean**
F02	0.995			0.988		
F14 – Observed	0.990	−0.6		0.992	−0.3	
F14 - Predicted	0.996	0.1	+0.7	0.982	−1.4	−0.8
F14 – Predicted no covariance	0.917	−7.9	−7.4	0.887	−10.9	−10.3
F14 – Predicted along g_max_	1.153	15.9	+16.5	1.184	18.9	+19.6
**Tibia Length**	Standardized mean	% diff. over F02 mean	% diff. over F14 observed mean	Standardized mean	% diff. over F02	% diff. over F14 observed mean
F02	0.941			0.941		
F14 – Observed	1.036	10.1		1.037	10.2	
F14 - Predicted	1.110	18.0	+7.2	1.064	13.1	+2.7
F14 – Predicted no covariance	1.142	21.3	+10.2	1.104	17.3	+6.6
F14 – Predicted along g_max_	1.173	24.6	+13.2	1.143	21.5	+10.4

### Evolvability and evolutionary constraints

Figure 6 shows how much body mass and tibia length would be expected to change in the absence of genetic covariance [42] (Table 2). Specifically, the predicted gains in tibia length (and hence rate of length increase per generation) would have been 20-30% greater than predicted gains with covariance. This would have produced tibiae that are ~7-10% longer than the observed change in mean tibia length, i.e. a total increase of 17-21% over the population mean at F02. In contrast, under an evolutionary model in which there is no covariance between the traits, body mass would decrease substantially every generation, instead of the flat evolutionary trajectory predicted using the full **G** matrix in Equation 1 (Figure 6). Cumulatively, mean body mass would have decreased by 7.9% and 10.9% in Lines 1 and 2, respectively (Table 2).

Figure 6 also illustrates the hypothetical evolutionary changes in tibia length and body mass in a selective breeding scenario where these genetically correlated traits were under selection in the same direction. By setting the selection gradient values for body mass to be the same magnitude but positive, the new bivariate vectors of selection are at right angles to the original vectors, in other words more parallel to the first eigenvector of the **G** matrix. In this situation, predicted gains in tibia length would have been ~40-60% greater than predicted gains under the original selection gradients. This would have resulted in a tibia that was over 20% longer than tibia length measured at F02 in both lines (Table 2). Here again, the greatest effect of this different selection regime along the line of greatest genetic variance would have been on body mass, which would have witnessed an increase of 16-19% over F02 values in both Longshanks lines (Figure 6, Table 2)

## Discussion

### Evolutionary autonomy of tibia length

We used a selective breeding program in mice to determine the degree to which the genetic correlation between body mass and tibia length constrains the latter from changing under directional selection for increased length. Our results show that tibia length can change rapidly and independently from body mass. In 14 generations, tibia length increased by ~9.4-12.8% (Table 1) in selectively bred lines vs a control cohort, while body mass remained the same in all three lines (Figure 2). Our quantitative genetic analyses showed that in both Longshanks lines, phenotypic correlations were around 0.52, while genetic correlations were 0.4-0.48. Thus, roughly one fifth of the genetic variation in tibia length is tied to variation in body mass, leaving up to 80% of the genetic variance in tibia length “free” to evolve independently of body mass, which explains why it responded relatively rapidly to selection. It should be noted, however, that this independent evolution was associated with substantially stronger selection pressures on tibia length: the absolute values of the selection gradients (β’s) for tibia length were over twice as great as those acting on body mass. Thus, the fitness benefits of increased tibia length far outweighed the negative fitness effects of body mass.

**Figure 7:**
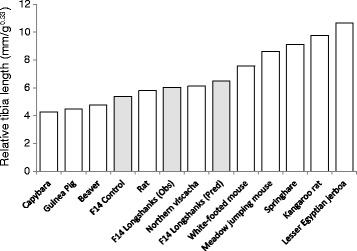
**Relative tibia length in selected rodents.** Relative tibia length is reported as tibia length (mm) divided by the cube root of body mass (g^0.33^). There is a progression from left to right from large-bodied rodents with relatively short tibiae (e.g., capybaras) to highly specialized bounding rodents with relatively long tibiae (e.g., jerboas and kangaroo rats). After 14 generations of selection, the Longshanks mouse approaches the relative tibia length of Northern viscachas (*Lagidium peruanum*) (see text) [15,49].

In fewer than 15 generations, artificial selection produced substantial changes in relative tibia length of ~3.5 units of phenotypic standard deviation, similar to other experiments in which the relationship between two phenotypic traits was the specific target of selection [21,34]. Such rapid phenotypic change is consistent with the rate and diversity of skeletal changes produced by selective breeding in domestic species, especially dog breeds [43,44], but also pigeons [45,46] and horses [47,48]. Our results can also be placed within the broader comparative context of limb skeletal evolution in rodents (Figure 7). In 14 generations, we produced mice in which relative tibia length exceeded that of rats, and was similar to that in Northern viscachas (*Lagidium peruanum*), a ~3 kg rabbit-like rodent known to hop in the rocky outcrops that form its habitat in Patagonia [49]. At F14, selection responses remain strong and, for the most part, linear (Figures 5 and 6). Assuming that selection pressures remain the same, and the **G**-matrix remains stable in the longer term [50], one can extrapolate future changes in the two traits (Additional file 8: Table S8) [21]. Under this selection regime, in Longshanks Line 1, it would take fewer than 100 generations to produce mice with body masses and tibia lengths roughly equivalent to kangaroo rats (*Dipodomys merriami*), a habitually bipedal desert rodent known for its hopping behavior [51,52]. Simply put, the locomotor skeletal system of rodents can evolve remarkably rapidly, much like the vertebrate skull [43,46] and overall skeletal size [48].

### Evolutionary constraints on tibia length

We evaluated the extent to which the genetic correlation with body mass constrains independent change in tibia length by simulating two evolutionary scenarios with selection and quantitative genetic parameters derived from the observed data. First, we assumed that the two traits had the same effect on fitness, but there was no genetic covariance between them [42,53]. In this scenario, the gains in tibia length would have been a third greater than predicted gains using a full **G** matrix. Through the existence of a positive genetic correlation, selection against increases in body mass has reduced the actual selective gains in tibia length, and by the same token the *rate* of evolutionary change in tibia length. In a comparative framework, the hypothetical increase in the rate of evolution of tibia length due to the absence of genetic correlation would produce relative tibia length equivalent to the kangaroo rat in fewer than 40 generations (Figures 6 and 7, Additional file 8: Table S8). Of course, this faster rate of evolution is achieved not only through the greater increase in tibia length, but also because body mass decreases rapidly when not genetically correlated with tibia length (see below). When extrapolated over more generations, this simulation leads to an unlikely scenario in which body mass becomes smaller than 5 g (Additional file 8: Table S8).

In the second scenario, we simulated evolutionary pressures along the line of greatest genetic variance with a full **G** matrix. Not surprisingly, in this situation the positive genetic correlation between body mass and tibia length favors rapid change in both traits, with tibia length increasing 60% more rapidly than in the observed and predicted data under selection gradients operating at near-right angles to **g**_**max**_. As in the first scenario, this scenario reveals that body mass would have changed as well, this time increasing by 20% in both lines. All else being equal, this type of selection regime could theoretically produce rat-sized mice in ~60 generations (~230 g, [54]). In a now-classic selection experiment, H.D. Goodale selected mice for increased body mass [55]. The experiment continued for over a 100 generations, producing the LG/J inbred mouse [56]. After ~35 generations, during which body mass had increased by 70%, the mice ceased to respond to selection, well short of a rat’s size. A limit to selection was probably reached because of the small founder population and hence lack of genetic variance (16 individuals [56]).

### Evolutionary constraints on body mass

One important outcome of this study concerns the theoretical changes in body mass under an evolutionary model with no additive genetic covariance between the traits [42]. We showed that the genetic correlation with body mass effectively slowed the rate of change in tibia length by ~30%. What this analysis also reveals, however, is that this correlation simultaneously prevents mean body mass from decreasing under selection. Judging by the relatively strong and negative selection gradients (β) for body mass (Additional file 3: Table S3), larger body masses had a negative impact on fitness, and should have been selected against. Yet larger individuals clearly remained in the gene pool over 14 generations of selection (Figures 2 and 6), suggesting that evolutionary changes in body mass are also constrained, and mean body mass is “carried along” as a correlated response to selection acting on tibia length (see also [11,12,57]). Thus, the constraint goes both ways: selection for independent increases in tibia length imposed a constraint that maintained mean body mass constant, despite strong selective pressures towards a decrease in the population means for that trait.

### Potential genetic basis of independent evolution in body mass and tibia length

Independent evolutionary change in tibia length is possible because a substantial portion of the genetic variation in tibia length is not associated with variation in body mass. This raises the question of the nature and relationship of the genetic loci that contribute to phenotypic variation in both traits, and whose allelic frequencies are changing under selection. Body mass and bone length are complex polygenic traits determined by dozens, if not hundreds of additive genetic loci [58-62]. Some of the genetic loci likely influence one trait or the other exclusively, while other loci have pleiotropic, and largely parallel, effects on both [62]. It is likely that at least some alleles at tibia-specific loci have changed in frequency. However, the fact that mean body mass does not decrease despite having a considerable negative impact on fitness suggests that pleiotropic loci for body mass and bone length are also involved in the response to selection of both traits.

One model that could explain the observed responses in both traits is differential epistasis [62]. In this model, it is not only allelic frequencies at the pleiotropic loci themselves that are changing, but also at epistatic loci that modify the effects of these pleiotropic loci on both traits (loci known as relationship QTLs or rQTLs [62]). Such loci would decrease the genetic/phenotypic correlation between the traits, in so doing modifying the allometric relationship between body mass and tibia length in the Longshanks and Control lines. For example, an rQTL could differentially increase the downstream effect of a pleiotropic locus on tibia length in the Longshanks mouse, without modulating the magnitude of its effect on body mass. This would maintain the constraining effect of the pleiotropic locus on both traits in both lines, while enabling the observed increases in tibia length in the Longshanks lines. In the absence of any pleiotropic or epistatic loci for these traits (i.e., in the absence of a genetic correlation, Figure 6), both would become “free” to respond to selection through allelic changes at trait-specific loci. In future experiments, we will seek to identify the nature, genomic location and phenotypic effects of single nucleotide polymorphisms whose frequencies have changed under artificial selection in this unique mouse sample.

## Conclusion

We have demonstrated, in vivo, and in real time, the impact of genetic correlations on the independent evolution of body mass and skeletal size in a mammalian model organism. This artificial selection experiment shows that independent changes in these complex traits are possible, and can be quite rapid. Our results suggest that the frequent and convergent evolution of relatively longer hind limbs among rodents [63], for example in the context of adaptive radiations and niche partitioning in heterogeneous environments [64,65], may also have occurred very rapidly on a geological timescale. Our simulated evolution under different selection and quantitative genetic scenarios also indicate, however, that this independent evolution is to some extent constrained by a genetic correlation. Specifically, our data show that the rate of independent evolution of tibia length is impeded by its correlation with body mass (and vice-versa), and that selection along the line of greatest genetic variance would allow for the most rapid, but commensurate, changes in body mass and tibia length.

## Methods

### Mouse stocks

All animal procedures have been approved by the Health Sciences Animal Care Committee at the University of Calgary (protocol AC13-0077). The mouse stock chosen for this artificial selection experiment was the Hsd-ICR (CD-1®) stock, a general purpose outbred laboratory model. Mice derived from this stock have previously been used in artificial selection experiments (e.g., [11,66]). All mice were obtained from colonies A and B from barrier 217 at Harlan Biosciences (Indianapolis, IN), and each mouse came from different parents. Three closed lines of mice were set up, two experimental lines (the Longshanks mice) in which mice were selectively bred (Lines 1 and 2), and one control line in which mice were bred at random (Line C). In each line, 16 founding breeding pairs were established. Mice were housed in individually ventilated cages (Greenline Sealsafe PLUS, Buguggiate, Italy), kept on a 12-hour light/dark cycle and a constant room temperature of 20°C, with food (Pico-Vac Mouse Diet 5061, LabDiet, St. Louis, MO) and water provided *ad libitum.*

### Breeding protocol and phenotyping

#### Selected lines

In each generation, after successful breeding, males were removed from the breeding pairs. Litters remained with their mothers until weaning at three weeks of age. Litter size was recorded, and male and female pups were then placed in separate cages with a maximum of five individuals per cage. At eight weeks old (+/− three days), all mice in Lines 1 and 2 were phenotyped and ranked within family to select the breeders for the following generation. The phenotypic assessment was performed as follows. Mice were anesthetized with a Ketamine/Xylazine solution (200 mg/10 mg per kg body mass), and weighed to the nearest 0.01 g. Mice were then positioned ventral side up on a clear plastic tray, with their hind limbs secured to the tray in an abducted position using 3 M Transpore© surgical tape. Next, a lead apron was placed over the lower abdomen, and the mice were radiographed (35 pKV, 3 mA, scan time ~20 seconds) in a cabinet X-ray system (Faxitron 43855A, Faxitron X-Ray Corp, Wheeling, IL). Digital images of the x-rays were acquired at a resolution of 12 pixels/mm using a digital X-ray scanner placed inside the cabinet (EZ240 Digital X-ray Scanner, NTB, Germany) (Additional file 9: Figure S1).

The digital images of the tibiae were landmarked using TPSDig2 v2.1 [67]. The first landmark was placed at the midpoint on the cranial border of the tibial epiphysis, and the second at the tip of the medial malleolus (Additional file 9: Figure S1). Tibia length was determined by calculating the distance between the two landmarks. To assess measurement error for the X-ray and landmarking, four mice (two males, two females) in generation F02 were measured by CR one after the other using the protocol described above, and the process was repeated five times. The mean relative standard error for the four mice was 0.1% (range: 0.08-0.13%). Furthermore, the mean intra-individual variance represented ~3.3% of the total variance, indicating a high signal-to-noise ratio. Thus, measurement error was deemed not to have a significant effect on tibia length measurements.

Next, breeders from each family were identified. The goal of the selective breeding protocol was to increase tibia length while maintaining body mass constant between the Longshanks and control mouse lines. Selecting for increases in tibia length independent of body mass ideally requires a regression-based approach, to identify individuals with the greatest positive residual tibia length after regressing on the cube root of body mass within sex. The relationship of body mass to tibia length in a given family was not always significant, however, because litter size, which varied from 2–18 individuals, and/or composition by sex, precluded the estimation of a linear regression within sex (e.g., some families had only 1–2 individuals of one sex). Instead, we used a ranking system in which males and females were ranked separately. Individuals were first ranked by absolute tibia length, and then again by relative tibial length, i.e., tibia length divided by (body mass)^0.33. The final rank of an individual was determined by summing the two ranks. This dual ranking system was used because either measure on its own is not sufficient to ensure mass-independent changes in bone length. For example, using absolute tibia length alone would likely result in selection for overall larger animals, given the genetic correlation between the traits. Similarly, relative tibia length is a ratio, and hence it is not possible to assess whether the traits being selected are increased tibia length (numerator), decreased body mass (denominator), or both. The summed rank controls for the effect of variation in body mass among siblings, while weighting the rank towards individuals with absolutely longer tibiae.

The top ranked male and female were selected as breeders. In the case of ties, the individual with the highest rank for relative tibia length was chosen as the breeder. The second highest ranked male and female from each family were also identified in case some families failed to produce litters within a generation. The breeders in a given family were paired randomly with breeders from two other families within an experimental line, i.e., disallowing sib mating and maximizing outbreeding. 16 pairs were set up each generation. Individuals who were not selected as breeders were euthanized 1–2 days after the phenotypic assessment by CO_2_ inhalation. Breeders were also euthanized by CO_2_ inhalation, in the case of sires once the dams were visibly pregnant, and in the case of dams once their litters were weaned (21 days postpartum). All carcasses were kept and stored at −20°C.

#### Control line

In Line C, experimental protocols were modified after generation F11. In the first 11 generations, control individuals were radiographed by placing the entire cage without its lid in the x-ray cabinet. Individual breeders were then selected at random from each control family. Breeding pairs were set up as in the selected lines, disallowing sib mating. To provide baseline data for comparison against the selected lines, subsamples of control mice were subjected to the full phenotypic assessment protocol in generations F04, F07 and F09. The full control cohort in generation F10 was measured as described above. As of generation F12, a subsample of three males and three females from each Line C family were subjected to this protocol, providing a continuous control baseline moving forward.

### Data analysis

#### Phenotypic changes due to selection

Changes due to selection on tibia length and body mass were analyzed using the General Linear Models package in Statistica v. 8.0 (StatSoft Inc, Tulsa, OK). For both variables, an analysis of covariance (ANCOVA) was performed, using tibia length or the cube root of body mass as response variables, sex, generation and line (Lines 1, 2 and C) as categorical factors, including their potential interaction, and age at measurement (in days) and litter size as covariates. The average sample size of a given group in the Longshanks lines (e.g., Line 1 females from F14) was 82.0 (range: 50–109), while in the Control line the average was 46.0 (range: 22–104) (see Additional file 1: Table S1). We also ran an ANCOVA to compare the trait means at F14 only, using sex and line as fixed factors. Results are reported as least squared (LS) means by generation, line and sex, computed for the covariates at their means.

#### Selection parameters

Cube root body mass and tibia length data were used to obtain selection parameters, including the directional selection differentials (s), selection coefficients (β), **G**-matrices and narrow-sense heritabilities (h^2^). In each line, cube root body mass and tibia length were first mean-standardized to facilitate comparisons of selection and evolvability estimates for continuous traits with different means and variances [26]. Moreover, generation F01 for both Lines 1 and 2 was not used to estimate the selection parameters, nor in any subsequent evolvability analysis, for two reasons. First, due to unforeseen circumstances, tibia length in F01 was measured at 11 weeks instead of eight. Second, F01 mice were fed a diet with higher fat content (~9% fat content, Pico-Vac Mouse Diet 5062, LabDiet, St. Louis, MO) designed for breeding colonies requiring higher energy levels for multiple litters. In subsequent generations, the mice were moved to a normal diet designed for single-litter breeding colonies (5% fat content, Pico-Vac Mouse Diet 5061). As a result of their older age and higher body mass, the inclusion of mice from generation F01 skews selection parameters, including the **G**-matrix, where it produces artificially high levels of environmental variance.

- Selection differentials, coefficients and evolutionary responses

Directional selection differentials (denoted ‘s’) were obtained by subtracting, in each generation, the vector of means of the individuals before selection, from the vector of means of the individuals after selection (i.e., mean body mass and tibia length for the 32 breeders). The cumulative selection differential was obtained by summing across generations. To test for the linearity of the selection response in tibia length, we regressed mean tibia length on its cumulative selection differential using linear only and linear plus quadratic terms [11,21]. The selection coefficients, *β*’s, known as selection gradients, are a set of partial regression coefficients (i.e., slopes) of relative fitness on the traits which provide an estimate of the magnitude and direction of selection acting on each trait. Raw selection coefficients were obtained by regressing relative fitness on the two traits. The observed vectors of selection responses, $\varDelta \overline{z}$, were obtained in each generation by subtracting the bivariate vector of trait means of the previous generation from the current generation. Total changes in tibia length and body mass were obtained by summing across generations. Note that there is no observed selection response for the last generation, F14.

b. **G**-matrices

We estimated the matrix of additive genetic variances and covariances (the **G**-matrix) for each Longshanks line separately using linear mixed effects models, also known as “animal models” [68]. **G**-matrices were estimated using restricted maximum likelihood as implemented in the program VCE (v. 6.0, [69,70]). Ideally, the **G**-matrix for each pair of successive generations should be used to determine short term responses to selection. However, in several instances, estimation of **G**-matrices over two (or even three) generations using the animal model produced unreliable or indeterminate matrices. At the same time, the estimate of the **G**-matrix using the entire pedigree could be biased by the patterns of phenotypic variance in later generations, which are the result of intense directional selection. As a compromise, we estimated **G**-matrices using the pedigree of the first five generations (F02-F06) for each line separately, under the assumption that the **G**-matrix would be relatively stable at such short evolutionary timescales. The animal models were run as linear regressions, with mean-standardized tibia length and body mass as dependent variables, age at measurement and litter size as continuous variables (covariates) and sex and animal ID as fixed effects.

### Estimation of evolvability and evolutionary constraints

A number of multivariate tools have been proposed to estimate how much the structure of the **G-**matrix influences the ability of a multivariate phenotype to respond to selection [26,42,71]. All are based on Equation 1, and measure the extent to which a phenotype responds in a direction and magnitude consistent with the direction and magnitude of selection, under the constraints imposed by the **G**-matrix. We used two simple measures of the impact of the genetic correlation between body mass and tibia length on the independent evolution of the latter.

First, we used Equation 1 to obtain the cumulative predicted changes in tibia length and body mass when the additive genetic covariance between the two was removed (i.e., the off-diagonal elements of **G** were set to zero) [42,53]. This measure illustrates how much and how fast each trait would change under directional selection if they were not genetically correlated. By obtaining the ratio of the predicted (or observed) changes under a **G**-matrix with no covariance to those using the full G-matrix, we obtained an estimate of how much the genetic correlation between the two traits has slowed selective gains in tibia length. This measure also provides an estimate of the effect of the genetic correlation on evolutionary changes in body mass.

The second and related measure is to obtain the cumulative predicted changes in tibia length and body mass using Equation 1, but when selection occurs parallel (or nearly so) to **g**_**max**_. This can be approximated by setting the selection gradients acting on each trait to have the same sign, in this case positive. This measure models the rate of change along directions that are more parallel to the adaptive line of least resistance. Taken together, these two measures illustrate how much the genetic correlation between the two traits affects change along, and away from, the line of greatest genetic variance.

## Availability of supporting data

The data set supporting the results of this article is available in the Dryad repository, http://datadryad.org/review?doi=doi:10.5061/dryad.g9q79 [72].

## Additional files

Additional file 1: Table S1. Least square means and standard deviations of cube root body mass (BM) and tibia length (TL) by sex and line over the first 14 generations of the selective breeding program. Additional file 2: Table S2. Results of the ANCOVA for differences in the means of cube root body mass and tibia length as a function of the three categorical factors (generation, line and sex), and their interactions. Values in bold are significant at the p < 0.05 (*) and p < 0.001 (**) levels. Additional file 3: Table S3. Generational mean-standardized means, selection differentials (s) and selection gradients (β) in cube root body mass and tibia length for Lines 1 and 2. Additional file 4: Table S4. Additive genetic variance/covariance matrix for Line 1 (top row) and Line 2 (bottom row, shaded), estimated from generations F02-F06 in each line. Diagonals are variances, above the diagonal is the covariance, below the additive genetic correlation (bold). Standard errors of the estimates are in brackets. Additional file 5: Table S5. Dominance genetic variance/covariance matrix for Line 1 (top row) and Line 2 (bottom row, shaded), estimated from generations F02-F06 in each line. Diagonals are variances, above the diagonal is the covariance, below the dominance genetic correlation (bold). Standard errors of the estimates are in brackets. Additional file 6: Table S6. Environmental variance/covariance matrix for Line 1 (top row) and Line 2 (bottom row, shaded), estimated from generations F02-F06 in each line. Diagonals are variances, above the diagonal is the covariance, below the additive genetic correlation (bold). Standard errors of the estimates are in brackets. Additional file 7: Table S7. Phenotypic variance/covariance matrix for Line 1 (top row) and Line 2 (bottom row, shaded), estimated from generations F02-F06 in each line. Diagonals are variances, above the diagonal is the covariance, below the additive genetic correlation (bold). Additional file 8: Table S8. Theoretical changes in body mass, tibia length and relative tibia length over future generations of selection in Longshanks Line 1, assuming similar selection regimes and genetic variance/covariance structure. Additional file 9: Figure S1. Digital radiography setup. The diagram shows the anesthetized mouse in supine position, with the stifle extended and held in place by surgical tape. The dashed lines show the region of interest (ROI) detected by the digital scanner. The grey area is the lead apron covering the lower abdominal and genital areas. The inset shows the resulting digital scan as well as the markers placed on the anatomical landmarks used to measure tibia length (solid line). Diagram and inset are not to scale.
