# Supplementary material for: Impacts of genetic correlation on the independent evolution of body mass and skeletal size in mammals
Source: BMC Evol Biol. 2014 Dec 14;14:258. doi: 10.1186/s12862-014-0258-0 (PMC4269856; doi:10.1186/s12862-014-0258-0)
Supplement: Additional file 1: Table S1. — Least square means and standard deviations of cube root body mass (BM) and tibia length (TL) by sex and line over the first 14 generations of the selective breeding program. [file 12862_2014_258_MOESM1_ESM.docx]

**Table S1**: Least square means and standard deviations of cube root body mass (BM) and tibia length (TL) by sex and line over the first 14 generations of the selective breeding program. These data are plotted in Figure 2.

| **Gen.** | **Sex** | **Line** | **N** | **LS Means - BM** | **SD - BM** | **LS Means - TL** | **SD - TL** |
| --- | --- | --- | --- | --- | --- | --- | --- |
| **F01** | **F** | 1 | 100 | 3.306 | 0.130 | 17.951 | 0.651 |
|  |  | 2 | 83 | 3.376 | 0.166 | 17.821 | 0.830 |
|  |  | C | - |  |  |  |  |
|  | **M** | 1 | 91 | 3.529 | 0.131 | 18.051 | 0.655 |
|  |  | 2 | 66 | 3.561 | 0.156 | 18.017 | 0.779 |
|  |  | C | - |  |  |  |  |
| **F02** | **F** | 1 | 105 | 3.225 | 0.109 | 18.094 | 0.544 |
|  |  | 2 | 105 | 3.241 | 0.108 | 17.978 | 0.539 |
|  |  | C | - |  |  |  |  |
|  | **M** | 1 | 101 | 3.450 | 0.109 | 18.244 | 0.544 |
|  |  | 2 | 89 | 3.436 | 0.108 | 18.230 | 0.539 |
|  |  | C | - |  |  |  |  |
| **F03** | **F** | 1 | 109 | 3.239 | 0.109 | 18.465 | 0.547 |
|  |  | 2 | 102 | 3.236 | 0.115 | 18.403 | 0.574 |
|  |  | C | - |  |  |  |  |
|  | **M** | 1 | 91 | 3.453 | 0.108 | 18.757 | 0.542 |
|  |  | 2 | 109 | 3.479 | 0.114 | 18.631 | 0.572 |
|  |  | C | - |  |  |  |  |
| **F04** | **F** | 1 | 94 | 3.193 | 0.114 | 18.520 | 0.572 |
|  |  | 2 | 75 | 3.173 | 0.107 | 18.321 | 0.534 |
|  |  | C | 33 | 3.145 | 0.109 | 17.523 | 0.544 |
|  | **M** | 1 | 97 | 3.388 | 0.114 | 18.781 | 0.573 |
|  |  | 2 | 71 | 3.430 | 0.107 | 18.548 | 0.535 |
|  |  | C | 30 | 3.387 | 0.109 | 17.756 | 0.543 |
| **F05** | **F** | 1 | 92 | 3.193 | 0.107 | 18.759 | 0.537 |
|  |  | 2 | 85 | 3.200 | 0.108 | 18.621 | 0.539 |
|  |  | C | - |  |  |  |  |
|  | **M** | 1 | 103 | 3.433 | 0.107 | 19.145 | 0.537 |
|  |  | 2 | 98 | 3.421 | 0.108 | 18.886 | 0.540 |
|  |  | C | - |  |  |  |  |
| **F06** | **F** | 1 | 64 | 3.179 | 0.107 | 18.874 | 0.536 |
|  |  | 2 | 92 | 3.251 | 0.107 | 18.691 | 0.534 |
|  |  | C | - |  |  |  |  |
|  | **M** | 1 | 83 | 3.401 | 0.107 | 19.191 | 0.536 |
|  |  | 2 | 83 | 3.460 | 0.107 | 19.088 | 0.534 |
|  |  | C | - |  |  |  |  |
| **F07** | **F** | 1 | 73 | 3.297 | 0.107 | 19.380 | 0.535 |
|  |  | 2 | 69 | 3.297 | 0.107 | 19.168 | 0.536 |
|  |  | C | 44 | 3.280 | 0.108 | 18.058 | 0.540 |
|  | **M** | 1 | 97 | 3.489 | 0.107 | 19.716 | 0.535 |
|  |  | 2 | 72 | 3.475 | 0.107 | 19.437 | 0.536 |
|  |  | C | 37 | 3.522 | 0.108 | 18.233 | 0.539 |
| **F08** | **F** | 1 | 86 | 3.228 | 0.107 | 19.312 | 0.538 |
|  |  | 2 | 88 | 3.289 | 0.107 | 19.235 | 0.534 |
|  |  | C | - |  |  |  |  |
|  | **M** | 1 | 81 | 3.409 | 0.107 | 19.412 | 0.536 |
|  |  | 2 | 80 | 3.485 | 0.107 | 19.559 | 0.534 |
|  |  | C | - |  |  |  |  |
| **F09** | **F** | 1 | 69 | 3.222 | 0.107 | 19.436 | 0.537 |
|  |  | 2 | 67 | 3.270 | 0.109 | 19.177 | 0.544 |
|  |  | C | 26 | 3.265 | 0.107 | 17.861 | 0.533 |
|  | **M** | 1 | 83 | 3.416 | 0.108 | 19.578 | 0.539 |
|  |  | 2 | 67 | 3.451 | 0.109 | 19.518 | 0.543 |
|  |  | C | 22 | 3.437 | 0.107 | 17.875 | 0.534 |
| **F10** | **F** | 1 | 80 | 3.198 | 0.109 | 19.347 | 0.543 |
|  |  | 2 | 74 | 3.243 | 0.108 | 19.166 | 0.543 |
|  |  | C | 104 | 3.247 | 0.109 | 17.713 | 0.547 |
|  | **M** | 1 | 74 | 3.382 | 0.108 | 19.571 | 0.541 |
|  |  | 2 | 74 | 3.432 | 0.108 | 19.408 | 0.543 |
|  |  | C | 93 | 3.469 | 0.109 | 17.834 | 0.544 |
| **F11** | **F** | 1 | 60 | 3.208 | 0.107 | 19.434 | 0.538 |
|  |  | 2 | 83 | 3.248 | 0.107 | 19.305 | 0.537 |
|  |  | C | - |  |  |  |  |
|  | **M** | 1 | 84 | 3.406 | 0.108 | 19.688 | 0.540 |
|  |  | 2 | 75 | 3.446 | 0.107 | 19.586 | 0.537 |
|  |  | C | - |  |  |  |  |
| **F12** | **F** | 1 | 66 | 3.228 | 0.108 | 19.609 | 0.540 |
|  |  | 2 | 80 | 3.247 | 0.107 | 19.514 | 0.535 |
|  |  | C | 43 | 3.167 | 0.108 | 17.177 | 0.542 |
|  | **M** | 1 | 61 | 3.410 | 0.108 | 19.854 | 0.543 |
|  |  | 2 | 78 | 3.406 | 0.107 | 19.689 | 0.535 |
|  |  | C | 38 | 3.349 | 0.108 | 17.396 | 0.541 |
| **F13** | **F** | 1 | 75 | 3.239 | 0.107 | 19.797 | 0.537 |
|  |  | 2 | 85 | 3.185 | 0.107 | 19.416 | 0.533 |
|  |  | C | 41 | 3.118 | 0.109 | 17.212 | 0.547 |
|  | **M** | 1 | 74 | 3.392 | 0.107 | 19.945 | 0.537 |
|  |  | 2 | 81 | 3.399 | 0.107 | 19.771 | 0.533 |
|  |  | C | 42 | 3.329 | 0.109 | 17.390 | 0.548 |
| **F14** | **F** | 1 | 73 | 3.191 | 0.107 | 19.764 | 0.535 |
|  |  | 2 | 43 | 3.161 | 0.107 | 19.143 | 0.537 |
|  |  | C | 21 | 3.188 | 0.107 | 17.608 | 0.536 |
|  | **M** | 1 | 65 | 3.387 | 0.107 | 19.966 | 0.535 |
|  |  | 2 | 66 | 3.364 | 0.108 | 19.702 | 0.541 |
|  |  | C | 21 | 3.392 | 0.107 | 17.824 | 0.536 |
