# Supplementary material for: Impacts of genetic correlation on the independent evolution of body mass and skeletal size in mammals
Source: BMC Evol Biol. 2014 Dec 14;14:258. doi: 10.1186/s12862-014-0258-0 (PMC4269856; doi:10.1186/s12862-014-0258-0)
Supplement: Additional file 2: Table S2. — Results of the ANCOVA for differences in the means of cube root body mass and tibia length as a function of the three categorical factors (generation, line and sex), and their interactions. Values in bold are significant at the p < 0.05 (*) and p < 0.001 (**) levels. [file 12862_2014_258_MOESM2_ESM.docx]

**Table S2**: Results of the ANCOVA for differences in the means of cube root body mass and tibia length as a function of the three categorical factors (generation, line and sex), and their interactions. Values in bold are significant at the p<0.05 (*) and p<0.001 (**) levels.

|  |  | **Cube Root Body Mass** | | **Tibia Length** | |
| --- | --- | --- | --- | --- | --- |
| Factor | **df** | **MS** | **F** | **MS** | **F** |
| Generation | 6 | 0.601 | **52.9**** | 22.079 | **77.61**** |
| Line | 1 | 0.429 | **37.8**** | 28.162 | **98.99**** |
| Sex | 0 | - | - | - | - |
| Generation*Line | 19 | 0.095 | **8.3**** | 6.002 | **21.09**** |
| Generation*Sex | 6 | 0.027 | **2.4*** | 0.212 | 0.74 |
| Line*Sex | 1 | 0.001 | 0.12 | 1.291 | **4.54*** |
| Generation*Line*Sex | 19 | 0.016 | 1.4 | 0.284 | 0.99 |
| Residual | 5157 | 0.011 |  | 0.285 |  |
