# Supplementary material for: Impacts of genetic correlation on the independent evolution of body mass and skeletal size in mammals
Source: BMC Evol Biol. 2014 Dec 14;14:258. doi: 10.1186/s12862-014-0258-0 (PMC4269856; doi:10.1186/s12862-014-0258-0)
Supplement: Additional file 3: Table S3. — Generational mean-standardized means, selection differentials (s) and selection gradients (β) in cube root body mass and tibia length for Lines 1 and 2. [file 12862_2014_258_MOESM3_ESM.docx]

| **LINE 1** | **Cube root body mass (g^0.33^)** | | | **Tibia length (mm)** | | |
| --- | --- | --- | --- | --- | --- | --- |
|  | Mean | s | β | Mean | s | β |
| F02 | 0.995 | 0.005 | -11.380 | 0.941 | 0.021 | 63.261 |
| F03 | 1.002 | 0.002 | -15.312 | 0.969 | 0.019 | 48.928 |
| F04 | 0.982 | -0.005 | -22.156 | 0.962 | 0.019 | 47.782 |
| F05 | 0.993 | -0.003 | -23.261 | 0.983 | 0.024 | 48.447 |
| F06 | 1.002 | -0.002 | -17.053 | 0.998 | 0.021 | 39.893 |
| F07 | 1.027 | -0.014 | -25.222 | 1.021 | 0.012 | 44.199 |
| F08 | 1.001 | 0.003 | -10.185 | 1.011 | 0.019 | 28.211 |
| F09 | 1.006 | 0.001 | -17.474 | 1.020 | 0.022 | 45.768 |
| F10 | 0.995 | 0.000 | -16.602 | 1.017 | 0.021 | 37.886 |
| F11 | 1.005 | -0.005 | -16.706 | 1.023 | 0.018 | 37.519 |
| F12 | 1.003 | 0.000 | -11.100 | 1.031 | 0.019 | 34.473 |
| F13 | 1.004 | 0.002 | -14.967 | 1.040 | 0.018 | 36.874 |
| F14 | 0.990 | -0.003 | -13.776 | 1.036 | 0.021 | 27.962 |
| **Mean** | **1.000** | **-0.001** | **-16.553** | **1.004** | **0.019** | **41.631** |
| **Cumulative** | **-** | **-0.017** | **-215.193** | **-** | **0.253** | **541.203** |
| **LINE 2** | **Cube root body mass (g^0.33^)** | | | **Tibia length (mm)** | | |
|  | Mean | s | β | Mean | s | β |
| F02 | 0.988 | 0.005 | -18.385 | 0.941 | 0.018 | 67.673 |
| F03 | 1.007 | -0.008 | -19.719 | 0.975 | 0.017 | 52.402 |
| F04 | 0.988 | -0.002 | -9.864 | 0.968 | 0.017 | 43.494 |
| F05 | 0.988 | -0.006 | -20.425 | 0.978 | 0.014 | 45.950 |
| F06 | 1.008 | -0.003 | -23.610 | 0.994 | 0.021 | 57.029 |
| F07 | 1.020 | -0.010 | -21.756 | 1.017 | 0.013 | 44.056 |
| F08 | 1.013 | 0.004 | -19.619 | 1.017 | 0.022 | 47.772 |
| F09 | 1.012 | 0.007 | -17.953 | 1.018 | 0.017 | 33.259 |
| F10 | 0.999 | 0.014 | -13.737 | 1.009 | 0.028 | 32.396 |
| F11 | 0.999 | 0.011 | -13.446 | 1.018 | 0.027 | 30.890 |
| F12 | 1.001 | -0.009 | -22.919 | 1.034 | 0.010 | 33.372 |
| F13 | 0.986 | 0.005 | -19.467 | 1.028 | 0.023 | 39.453 |
| F14 | 0.992 | -0.003 | -32.268 | 1.037 | 0.014 | 46.464 |
| **Mean** | **1.000** | **0.000** | **-19.474** | **1.003** | **0.018** | **44.170** |
| **Cumulative** | **-** | **0.002** | **-253.165** | **-** | **0.239** | **574.210** |

**Table S3**: Generational mean-standardized means, selection differentials (s) and selection gradients (β) in cube root body mass and tibia length for Lines 1 and 2.
