# Supplementary material for: Impacts of genetic correlation on the independent evolution of body mass and skeletal size in mammals
Source: BMC Evol Biol. 2014 Dec 14;14:258. doi: 10.1186/s12862-014-0258-0 (PMC4269856; doi:10.1186/s12862-014-0258-0)
Supplement: Additional file 4: Table S4. — Additive genetic variance/covariance matrix for Line 1 (top row) and Line 2 (bottom row, shaded), estimated from generations F02-F06 in each line. Diagonals are variances, above the diagonal is the covariance, below the additive genetic correlation (bold). Standard errors of the estimates are in brackets. [file 12862_2014_258_MOESM4_ESM.docx]

**Table S4**: Additive genetic variance/covariance matrix for Line 1 (top row) and Line 2 (bottom row, shaded), estimated from generations F02-F06 in each line. Diagonals are variances, above the diagonal is the covariance, below the additive genetic correlation (bold). Standard errors of the estimates are in brackets.

| **Additive** | Body Mass (x 10^-4^) | Tibia Length (x 10^-4^) |
| --- | --- | --- |
| Body Mass (x 10^-4^) | 3.90 (1.08) | 1.54 (0.65) |
|  | 4.57 (1.27) | 1.80 (0.65) |
| Tibia Length (x 10^-4^) | **0.397** | 3.91 (0.56) |
|  | **0.477** | 3.08 (0.47) |
