# Supplementary material for: Impacts of genetic correlation on the independent evolution of body mass and skeletal size in mammals
Source: BMC Evol Biol. 2014 Dec 14;14:258. doi: 10.1186/s12862-014-0258-0 (PMC4269856; doi:10.1186/s12862-014-0258-0)
Supplement: Additional file 5: Table S5. — Dominance genetic variance/covariance matrix for Line 1 (top row) and Line 2 (bottom row, shaded), estimated from generations F02-F06 in each line. Diagonals are variances, above the diagonal is the covariance, below the dominance genetic correlation (bold). Standard errors of the estimates are in brackets. [file 12862_2014_258_MOESM5_ESM.docx]

**Table S5**: Dominance genetic variance/covariance matrix for Line 1 (top row) and Line 2 (bottom row, shaded), estimated from generations F02-F06 in each line. Diagonals are variances, above the diagonal is the covariance, below the dominance genetic correlation (bold). Standard errors of the estimates are in brackets.

| **Dominance** | Body Mass (x 10^-4^) | Tibia Length (x 10^-4^) |
| --- | --- | --- |
| Body Mass (x 10^-4^) | 0.93 (1.52) | 0.88 (1.24) |
|  | 2.83 (3.14) | 1.14 (1.87) |
| Tibia Length (x 10^-4^) | **0.914** | 1.00 (1.25) |
|  | **0.523** | 1.68 (1.79) |
