# Supplementary material for: Impacts of genetic correlation on the independent evolution of body mass and skeletal size in mammals
Source: BMC Evol Biol. 2014 Dec 14;14:258. doi: 10.1186/s12862-014-0258-0 (PMC4269856; doi:10.1186/s12862-014-0258-0)
Supplement: Additional file 6: Table S6. — Environmental variance/covariance matrix for Line 1 (top row) and Line 2 (bottom row, shaded), estimated from generations F02-F06 in each line. Diagonals are variances, above the diagonal is the covariance, below the additive genetic correlation (bold). Standard errors of the estimates are in brackets. [file 12862_2014_258_MOESM6_ESM.docx]

**Table S6**: Environmental variance/covariance matrix for Line 1 (top row) and Line 2 (bottom row, shaded), estimated from generations F02-F06 in each line. Diagonals are variances, above the diagonal is the covariance, below the environmental correlation (bold). Standard errors of the estimates are in brackets.

| **Environment** | Body Mass (x 10^-4^) | Tibia Length (x 10^-4^) |
| --- | --- | --- |
| Body Mass (x 10^-4^) | 3.95 (0.67) | 1.69 (0.39) |
|  | 4.40 (0.80) | 1.62 (0.44) |
| Tibia Length (x 10^-4^) | **0.636** | 1.80 (0.33) |
|  | **0.615** | 1.57 (0.29) |
