# Supplementary material for: Impacts of genetic correlation on the independent evolution of body mass and skeletal size in mammals
Source: BMC Evol Biol. 2014 Dec 14;14:258. doi: 10.1186/s12862-014-0258-0 (PMC4269856; doi:10.1186/s12862-014-0258-0)
Supplement: Additional file 7: Table S7. — Phenotypic variance/covariance matrix for Line 1 (top row) and Line 2 (bottom row, shaded), estimated from generations F02-F06 in each line. Diagonals are variances, above the diagonal is the covariance, below the additive genetic correlation (bold). [file 12862_2014_258_MOESM7_ESM.docx]

**Table S7**: Phenotypic variance/covariance matrix for Line 1 (top row) and Line 2 (bottom row, shaded), estimated from generations F02-F06 in each line. Diagonals are variances, above the diagonal is the covariance, below the phenotypic correlation (bold).

| **Phenotypic** | Body Mass (x 10^-4^) | Tibia Length (x 10^-4^) |
| --- | --- | --- |
| Body Mass (x 10^-4^) | 8.78 | 4.12 |
|  | 11.81 | 4.56 |
| Tibia Length (x 10^-4^) | **0.538** | 6.71 |
|  | **0.526** | 6.34 |
