# Supplementary material for: Impacts of genetic correlation on the independent evolution of body mass and skeletal size in mammals
Source: BMC Evol Biol. 2014 Dec 14;14:258. doi: 10.1186/s12862-014-0258-0 (PMC4269856; doi:10.1186/s12862-014-0258-0)
Supplement: Additional file 8: Table S8. — Theoretical changes in body mass, tibia length and relative tibia length over future generations of selection in Longshanks Line 1, assuming similar selection regimes and genetic variance/covariance structure. [file 12862_2014_258_MOESM8_ESM.docx]

| **Full G-matrix** | Body Mass (g) | Tibia Length (mm) | Relative Tibia Length (mm/g^0.33^) |
| --- | --- | --- | --- |
| *F14 Observed* | *36.19* | *19.85* | *6.04* |
| F14 Predicted | 36.06 | 21.45 | 6.19 |
| F28 | 36.05 | 24.87 | 6.89 |
| F42 | 36.05 | 28.28 | 7.56 |
| F56 | 36.04 | 31.70 | 8.19 |
| F70 | 36.03 | 35.11 | 8.80 |
| F84 | 36.03 | 38.53 | 9.39 |
| F98 | 36.02 | 41.94 | 9.95 |
| **G-matrix no covariance** | Body Mass (g) | Tibia Length (mm) | Relative Tibia Length (mm/g^0.33^) |
| F14 Predicted | 27.70 | 22.09 | 7.30 |
| F28 | 20.73 | 26.15 | 9.52 |
| F42 | 15.04 | 30.20 | 12.23 |
| F56 | 10.51 | 34.25 | 15.64 |
| F70 | 6.99 | 38.31 | 20.04 |
| F84 | 4.36 | 42.36 | 25.94 |
| F98 | 2.49 | 46.41 | 34.26 |
| **Positive selection gradients** | Body Mass (g) | Tibia Length (mm) | Relative Tibia Length (mm/g^0.33^) |
| F14 Predicted | 57.55 | 22.73 | 5.89 |
| F28 | 86.20 | 27.42 | 6.21 |
| F42 | 123.07 | 32.12 | 6.46 |
| F56 | 169.20 | 36.81 | 6.66 |
| F70 | 225.60 | 41.50 | 6.82 |
| F84 | 293.33 | 46.19 | 6.95 |
| F98 | 373.42 | 50.89 | 7.07 |

**Table S8**: Theoretical changes in body mass, tibia length and relative tibia length over future generations of selection in Longshanks Line 1, assuming similar selection regimes and genetic variance/covariance structure.
