# Supplementary figures and images for: Impacts of genetic correlation on the independent evolution of body mass and skeletal size in mammals
Source: BMC Evol Biol. 2014 Dec 14;14:258. doi: 10.1186/s12862-014-0258-0 (PMC4269856; doi:10.1186/s12862-014-0258-0)

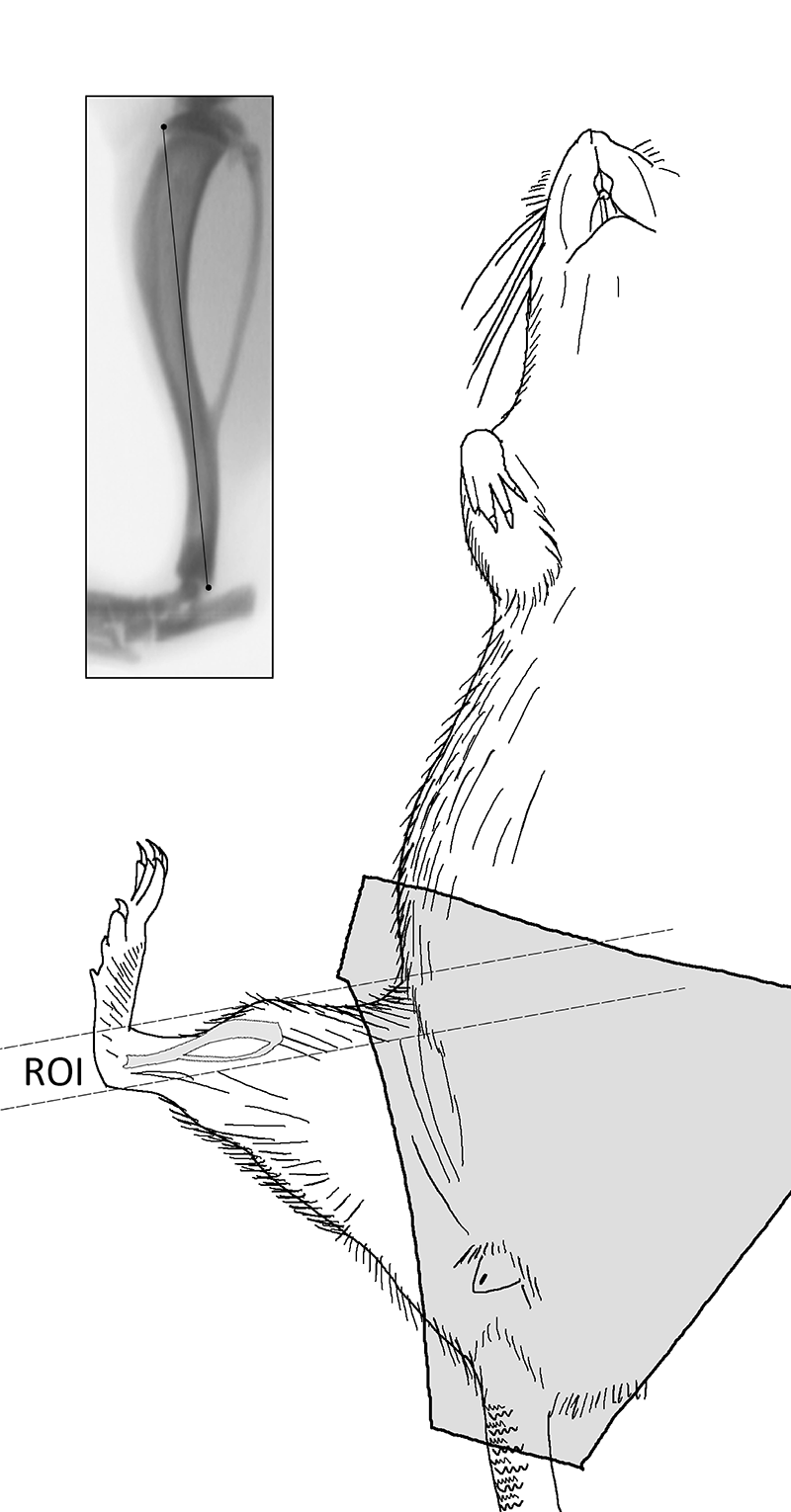

Supplement: Additional file 9: Figure S1. — Digital radiography setup. The diagram shows the anesthetized mouse in supine position, with the stifle extended and held in place by surgical tape. The dashed lines show the region of interest (ROI) detected by the digital scanner. The grey area is the lead apron covering the lower abdominal and genital areas. The inset shows the resulting digital scan as well as the markers placed on the anatomical landmarks used to measure tibia length (solid line). Diagram and inset are not to scale. [file 12862_2014_258_MOESM9_ESM.tiff]
